# Structural and Conformational Aspects in the Chemistry of Heterocycles

**DOI:** 10.3390/molecules25153461

**Published:** 2020-07-30

**Authors:** Bagrat A. Shainyan

**Affiliations:** A. E. Favorsky Irkutsk Institute of Organic Chemistry, Siberian Branch of the Russian Academy of Sciences, 1 Favorsky Street, 664033 Irkutsk, Russia; bagrat@irioch.irk.ru

Heterocyclic compounds represent more than half of all known organic compounds, so the growing interest in this field of chemistry is not surprising. Structure and conformations are a key factor determining the biological activity, including pharmaceutical properties, and reactivity of heterocycles. In this Special Issue, only several aspects have been discussed, the main focus being on the 14 group elements. The difference between the structure and conformational properties of heterocycles and those of their carbon predecessors is determined, first of all, by different bond distances and bond angles, the different electronegativity of carbon and heteroatoms and the presence of lone pairs. Thus, in the review of the Editor of this Special Issue on silacyclohexanes and sila(hetero)cyclohexanes as heterocycles with electropositive heteroatom [1], principally different structural and conformational features were demonstrated both in gas phase and in solution by GED, LT NMR, FT-IR spectroscopy, and theoretical calculations in comparison with corresponding cyclohexanes, oxanes, thianes and piperidines. The inversion of the role of steric, hyperconjugation, stereoelectronic, and electrostatic effects, as well as of conformational preferences, was clearly shown. Another type of the 14 group elements containing heterocycles was investigated theoretically in the paper on sila- and germatranes (tricyclic intramolecular complexes), their bi- and monocyclic analogues—sil- or germocanes and hyposilatranes and hypogermatranes [2]. A pivotal factor determining the properties of all compounds of this type is the intramolecular transannular N→X (X = Si, Ge) bond. The authors showed that the reactions with alcohols, as well as the reactions with water studied earlier by the same group, proceed via the heterocycle ring opening, with the rupture of the X–O bond but with retention of the transannular N→X bond. The activation energies and Gibbs activation energies decrease in the order atranes > ocanes > hypoatranes and Si > Ge. Even larger, 11-membered tetrel atoms (Si, Ge, Sn) containing heterocycles were synthesized and structurally investigated by a group of German chemists [3]. The Sn and Ge congeners were crystallized, but in quite different conformations which were attributed to different size and bond lengths with the tetrel atom. Thermogravimetric and differential scanning calorimetry analysis revealed that the thermal stability of the synthesized compounds decreases in the order Si > Ge > Sn.

A unique review by V. Kuznetsov [4] is devoted to drastic conformational changes occurring upon placing simple molecules inside the cavity of fullerenes or nanotubes. The review starts with incorporation of simple molecules, like alkanes, but includes also different heterocycles containing heteroatoms B, N, O, Si in various combinations. Briefly summarizing, the conformations which are unstable for isolated molecules (staggered alkanes, ‘perpendicular’ alkenes, twist or boat heterocycles) often become global minima for the incorporated molecules.

In the paper by Hungarian and German researchers [5], a synthetically challenging aminophenanthrol motif was successfully prepared on the example of 10-morpholinobenzyl-9-phenanthrol by the three-component reaction of 9-phenanthrol, benzaldehyde, and morpholine. The product was then involved in reactions with cyclic imines—3,4-dihydroisoquinoline, 6,7-dihydrothieno[3,2-c] pyridine, or 4,9-dihydro-β-carboline—and the structure of the formed products was determined by the detailed NMR analysis. This allowed them to conclude on the stereoselective formation of phenanthr[9,10-e][1,3] oxazines in the course of the [4 + 2] heterocyclization reaction.

Finally, in a somewhat specific work of K. Pihlaja et al. [6], the enthalpies of combustion of polymethylated 1,3-dioxanes were measured to estimate the Δ*H_f_* values in the gas phase. The latter values allowed the assessment of the energy difference between the chair and 2,5-twist conformers and the formation of a conclusion on the relative location of the methyl groups (pseudoequatorial, axial, diaxial, pseudoaxial, etc.) in the studied compounds.

As the Guest Editor, I believe that each of the above contributions will find its own readers. I do realize that the subject is far from being fully covered; for example, such interesting topics as conformations of heavier chalcogen- (Se, Te) or pnictogen-containing (P, As, Sb) heterocycles have been left outside of this Special Issue, leaving a room for new reviews and original articles in the field.
